# Activated Akt1 accelerates MMTV-c-ErbB2 mammary tumourigenesis in mice without activation of ErbB3

**DOI:** 10.1186/bcr2132

**Published:** 2008-08-13

**Authors:** Christian D Young, Erica C Nolte, Andrew Lewis, Natalie J Serkova, Steven M Anderson

**Affiliations:** 1 Department of Pathology, University of Colorado Denver, Research Complex I, South Tower, Mail Stop 8104, 12801 East 17thAvenue, Aurora, CO 80045, USA; 2 Program in Cancer Biology, University of Colorado Denver, Research Complex I, South Tower, Mail Stop 8104, 12801 East 17thAvenue, Aurora, CO 80045, USA; 3 Department of Anesthesiology, University of Colorado Denver, AO1, 12631 East 17thAvenue, Aurora, CO 80045, USA

## Abstract

**Introduction:**

ErbB2, a member of the epidermal growth factor receptor (EGFR) family, is overexpressed in 20% to 30% of human breast cancer cases and forms oncogenic signalling complexes when dimerised to ErbB3 or other EGFR family members.

**Methods:**

We crossed mouse mammary tumour virus (MMTV)-myr-Akt1 transgenic mice (which express constitutively active Akt1 in the mammary gland) with MMTV-c-ErbB2 transgenic mice to evaluate the role of Akt1 activation in ErbB2-induced mammary carcinoma using immunoblot analysis, magnetic resonance spectroscopy and histological analyses.

**Results:**

Bitransgenic MMTV-c-ErbB2, MMTV-myr-Akt1 mice develop mammary tumours twice as fast as MMTV-c-ErbB2 mice. The bitransgenic tumours were less organised, had more mitotic figures and fewer apoptotic cells. However, many bitransgenic tumours displayed areas of extensive necrosis compared with tumours from MMTV-c-ErbB2 mice. The two tumour types demonstrate dramatically different expression and activation of EGFR family members, as well as different metabolic profiles. c-ErbB2 tumours demonstrate overexpression of EGFR, ErbB2, ErbB3 and ErbB4, and activation/phosphorylation of both ErbB2 and ErbB3, underscoring the importance of the entire EGFR family in ErbB2-induced tumourigenesis. Tumours from bitransgenic mice overexpress the myr-Akt1 and ErbB2 transgenes, but there was dramatically less overexpression and phosphorylation of ErbB3, diminished phosphorylation of ErbB2, decreased level of EGFR protein and undetectable ErbB4 protein. There was also an observable attenuation in a subset of tyrosine-phosphorylated secondary signalling molecules in the bitransgenic tumours compared with c-ErbB2 tumours, but Erk was activated/phosphorylated in both tumour types. Finally, the bitransgenic tumours were metabolically more active as indicated by increased glucose transporter 1 (GLUT1) expression, elevated lactate production and decreased intracellular glucose (suggesting increased glycolysis).

**Conclusion:**

Expression of activated Akt1 in MMTV-c-ErbB2 mice accelerates tumourigenesis with a reduced requirement for signalling through the EGFR family, as well as a reduced requirement for a subset of downstream signaling molecules with a metabolic shift in the tumours from bitransgenic mice. The reduction in signalling downstream of ErbB2 when Akt is activated suggest a possible mechanism by which tumour cells can become resistant to ErbB2-targeted therapies, necessitating therapies that target oncogenic signalling events downstream of ErbB2.

## Introduction

The ErbB2/Neu/HER2 oncogene is amplified and overexpressed in 20% to 30% of human breast cancer cases, and expression of ErbB2 is associated with aggressive metastatic tumour behaviour, decreased time to clinical relapse and poor prognosis [1,2]. The importance of c-ErbB2 in mammary tumourigenesis was further established through a study of transgenic mice that expressed either activated ErbB2 (Neu-NT) [3,4] or non-activated c-ErbB2 [5,6]. Activated ErbB2-induced tumours in male and female mice have an average time to tumour appearance of 114 and 89 days, respectively [3], while expression of non-activated c-ErbB2 in the mammary gland of transgenic mice resulted in tumour formation in female mice in 150 to 300 days, with the latency depending on the founder line examined [5,6]. The most extensively investigated transgenic line to date is the mouse mammary tumour virus (MMTV)-c-ErbB2 line 202 female mice in which adenocarcinomas appear with an average latency of 205 days [6]. Overexpression of c-ErbB2 mRNA and protein, elevated c-ErbB2 kinase activity and increased tyrosine phosphorylation of cellular proteins was observed in tumour tissue, but not in normal mammary tissue from the same mouse [6].

Protein levels of ErbB3 and tyrosine phosphorylation of ErbB3 are increased in mammary tumours from transgenic mice expressing activated ErbB2 (Neu-DL) [7], suggesting that ErbB2 and ErbB3 function as an oncogenic unit [8,9]. Holbro *et al. *[9] demonstrated that loss of either functional ErbB2 or ErbB3 results in a loss of tumour cell proliferation even though ErbB3 does not possess an active tyrosine kinase domain [10]. ErbB3 phosphorylation activates phosphatidylinositol 3-kinase (PI3K) and its downstream target, Akt, thus providing a possible mechanism for the requirement for both ErbB2 and ErbB3 in stimulating mammary tumourigenesis. This gives rise to the hypothesis that expression of activated Akt could compensate for the expression of ErbB3 in ErbB2-induced mammary tumours.

Our group and others have demonstrated that expression of activated Akt1 [11,12] or overexpression of non-activated Akt1 [13] can delay mammary gland involution. In spite of the fact that Akt was discovered as an oncogene which induces leukaemia [14], mammary tumours were not observed in these transgenic mice [11-13]. Hutchinson *et al. *demonstrated that activated Akt1 could accelerate mammary tumourigenesis in transgenic mice that express activated ErbB2 [15]. Similarly, deletion of one or both alleles of phosphatase and tensin homolog (PTEN), a negative regulator of Akt signalling, accelerates tumour induction in another ErbB2 mouse mammary tumour model [16]. In the current study, we demonstrate that transgenic expression of activated Akt1 can accelerate mammary tumourigenesis in the MMTV-c-ErbB2 mice. However, we observe a significant attenuation of tyrosine kinase signalling in tumours from the bitransgenic MMTV-myr-Akt1, MMTV-c-ErbB2 animals compared with tumours from the MMTV-c-ErbB2 animals, particularly with regard to ErbB3 and Src. These results have implications for human ErbB2-positive tumours that may also have high levels of activated Akt, whether due to the loss of the tumour suppressor PTEN or mutations in either PI3K or Akt.

## Materials and methods

### Mice lines

The MMTV-c-ErbB2 (line 202) transgenic mice [6] were obtained from The Jackson Laboratory, Bar Harbor, ME. Details of the MMTV-myr-Akt1 mice have been previously described [11]. These two FVB-derived transgenic lines were crossed and progeny genotypes were determined by PCR analysis. Virgin MMTV-c-ErbB2 and bitransgenic MMTV-c-ErbB2, MMTV-myr-Akt1 female mice were palpated weekly to detect the presence of mammary tumours starting at 60 days of age. Tumours were excised when they reached 1 cm in diameter and 1 cm deep, and normal, non-tumourigenic mammary tissue was harvested from the same animal at the time of tumour harvest. All mice were maintained in the Center for Comparative Medicine at the University of Colorado Denver – Anshutz Medical Campus, an Association for Assessment and Accrediation of Laboratory Animal Care-approved facility, and used in accordance with Institutional Animal Care and Use Committee-approved protocols.

### Isolation of tail DNA and genotyping by PCR

DNA was extracted from 1.5 cm sections of tail and genotyping was performed using previously described protocols [11]. Detection of the Myr-Akt1 transgene utilised a forward primer which anneals to the Akt1 sequence: (5'-GCCGCTACTATGCCATGAAGA-3') and a reverse primer which anneals to the HA (haemagglutin) epitope: (5'-GTAATCTGGAACATCGTATGGGTA-3'). Detection of the ErbB2 transgene utilised the forward primer (Neu-3) 5'-CGGAACCCACATCAGGCC-3' and the reverse primer (Neu-4) 5'-TTTCCTGCAGCAGCCTACGC-3' [17].

### Mutation analysis of tumour *ERBB2 *transgene

Total RNA isolated from tumour samples was subjected to single-strand cDNA synthesis using 2.5 μM random hexamers and 1 μg RNA. Amplification of the rat *ERBB2 *gene, nucleotides 1492 to 2117, was performed using forward primer AB2913, 5'-CGGAACCCACATCAGGCC-3', and reverse primer AB1310, 5'-TTTCCTGCAGCAGCCTACGC-3', as previously described [18]. The PCR products were separated on a 2% agarose gel, the bands of interest (representing truncated *ERBB2*) were purified and then re-amplified using the same primers. Sequence analysis was conducted by the University of Colorado Cancer Center Sequencing Core using forward primer 1882, 5'-CACTACAAGGACTCGTCCT-3', and reverse primer 2133, 5'-CCAACGACCACCACTAAG-3'.

### Histological analysis and mitotic index quantification

Dissected tumours and normal mammary tissue were fixed in 4% neutral buffer formalin, embedded in paraffin, sectioned (4 μm) and stained with haematoxylin and eosin. Histological sectioning and staining were performed by the Histology Service, Department of Pathology, University of Colorado School of Medicine. The mitotic index of tumours of each genotype was determined by counting the number of mitotic figures in 10 fields of view with a magnification of 500 and the data were presented as the mean of three tumours +/- standard deviation (SD).

### Quantification of apoptotic cells

Detection of apoptotic cells was performed by immunohistochemical staining with anti-active caspase-3 antibody (Cell Signaling Technologies, Beverly, MA, USA). For antigen retrieval, slides in citrate buffer were heated in a microwave for 20 minutes and allowed to cool before blocking with 10% normal goat serum. Slides were incubated overnight at 4°C with anti-activated caspase-3 antibody at a dilution of 1:100. Non-specific peroxidase activity was quenched with 1% hydrogen peroxide followed by secondary antibody (goat anti-rabbit) then tertiary Vector ABC (Vector Laboratories, Burlingame, CA, USA). Colour development was achieved by incubation with DAB followed by counterstaining with Gill's Haematoxylin. Cell counts were performed on a minimum of five fields of view per slide from three mice (total cells counted ranged from 1800 to 2500 per mouse).

### Immunoblot analysis

Protein was extracted from frozen tumour tissue and normal tissue by grinding them to a powder under liquid nitrogen, resuspended in Mammary Gland Lysis Buffer (50 mM Tris (2-Amino-2-(hydroxymethyl)-1,3-propanediol) pH 7.4, 150 mM sodium chloride, 2 mM EDTA, 50 mM sodium fluoride, 1% Triton X-100, 1% deoxycholic acid, 0.1% sodium dodecyl sulfate [SDS], 1 mM dithiothreitol, 5 mM sodium orthovanadate, 100 μg/ml phenylmethanesulphonylfluoride and a complete protease inhibitor cocktail (Roche Applied Sciences, Indianapolis, IN, USA)), followed by Dounce homogenisation. Lysates were clarified by centrifugation and protein concentrations determined using the Bradford assay (BioRad, Hercules, CA, USA). Equal amounts of total protein per lane (2 to 50 μg) were resolved on SDS-polyacrylamide gels, transferred to polyvinylidene difluoride (PVDF) membrane (Immobilon-P, Millipore, Bedford, MA, USA) and immunoblotted with the desired antibody.

Anti-HA antibody was obtained from Roche Applied Sciences (Indianapolis, IN, USA). Anti-phospho-ErbB2 (Tyr877), anti-phospho-ErbB3 (Tyr1289), anti-phospho-retinoblastoma (Rb) (Ser780), anti-phospho-Akt (Ser473), anti-phospho-Src (Tyr416), anti-phospho-Gab2 (Tyr452), anti-phospho-Shc (Tyr313), anti-phospho-Shc (Tyr239/240), anti-Akt, anti-p15, anti-p27 and anti-cyclin D1 antibodies were obtained from Cell Signaling Technologies (Beverly, MA, USA). The anti-ErbB2, anti-ErbB3, anti-ErbB4, anti-EGFR, anti-Src, anti-Shc, anti-β-actin and anti-ERK1 antibodies were obtained from Santa Cruz Biotechnology (Santa Cruz, CA, USA). The anti-phospho-ErbB2 (Tyr1248) was obtained from Abcam (Cambridge, MA, USA). The anti-Rb antibody was obtained from BD Pharmingen (San Jose, CA, USA). The anti-phospho-ERK antibody was obtained from Promega (Madison, WI, USA). The anti-Gab2 polyclonal antibody was generously supplied by Dr. Haihua Gu (University of Colorado, Denver, USA). The glucose transporter 1 (GLUT1) polyclonal antibody was generated by Global Peptide (Fort Collins, CO, USA) by immunising rabbits with a peptide corresponding to the C-terminus of the human/mouse GLUT1 sequence (KTPEELFHPLGADSQV) and affinity purifying the resulting IgG.

### Metabolic profiling of tumours by magnetic resonance spectroscopy

Snap-frozen tissues were ground into a powder under liquid nitrogen then homogenised by sonication in chloroform-methanol to precipitate proteins and separate aqueous and lipid-soluble metabolites as described [19]. The lyophilised aqueous and lipid extracts were dissolved in deuterated solvents and analysed using high-resolution ^1^H-magnetic resonance spectroscopy (MRS) with the Bruker narrow-bore 500 MHz DRX system and an inverse TXI-5-mm probe (Bruker Biospin Inc., Fremont, CA, USA). The following ^1^H-nuclear magnetic resonance (NMR) parameters with water suppression ('zgpr') were used: 500.12 MHz operating ^1^H frequency; 40 accumulations; 0 dB power level; 55 dB irradiation power level; 7.5 μs pulse width; 12 ppm spectral width; and 12.8 second repetition time (fully relaxed). An external standard substance, trimethylsilyl propionic-2,2,3,3,-d_4 _acid ([TMSP] 20 and 50 mM in heavy water) in a thin sealed glass capillary was placed into the NMR tubes during all experiments for metabolite quantification and as a ^1^H chemical shift reference (at 0 ppm). After performing Fourier transformation, phase and base line corrections, each ^1^H peak of corresponding metabolites was integrated using 1D WINNMR program (Bruker Biospin Inc., Fremont, CA, USA). The NMR peak assignment was confirmed by two-dimensional NMR spectra [19]. All quantitative data sets from ^1^H-MRS are reported as mean ± SD (n = 5 for each group). The p values (from analysis of variance [ANOVA]) below 0.05 were considered as statistically significant.

## Results

### Activated myr-Akt1 accelerates mammary tumourigenesis in MMTV-c-ErbB2 transgenic mice

**Figure 1:**
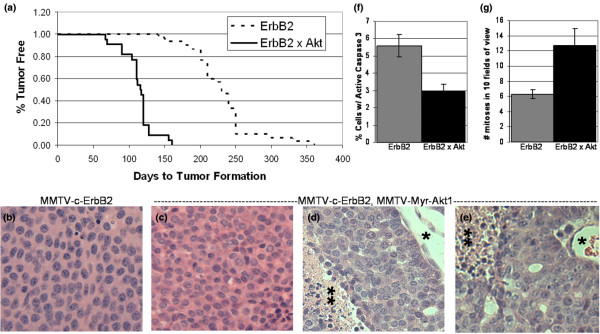
Bitransgenic MMTV-myr-Akt1, MMTV-c-ErbB2 mice have decreased mammary tumour latency, more aggressive tumour histology and decreased apoptosis compared with MMTV-c-ErbB2 mice. **(a) **Mammary tumour latency in bitransgenic MMTV-myr-Akt1, MMTV-c-ErbB2 mice and MMTV-c-ErbB2 mice. Sixty days after birth, bitransgenic MMTV-myr-Akt1, MMTV-c-ErbB2 and MMTV-c-ErbB2 mice were palpated weekly to monitor for the presence of mammary tumours. The graph shows the rate at which tumours were first detected for both genotypes. A total of 22 bitransgenic mice and 30 MMTV-c-ErbB2 mice were monitored and the graph shows the number of days to tumour detection versus the percentage of tumour-free mice. **(b-e) **Haematoxylin and eosin stained tumour sections. (b) Tumour derived from a MMTV-c-ErbB2 mouse. (c-e) Tumors derived from bitransgenic MMTV-myr-Akt1, MMTV-c-ErbB2 mice. (c) A bitransgenic tumour with histology similar to that of c-ErbB2 tumours. (d-e) Two different bitransgenic tumours demonstrating necrotic tumour tissue distal to a blood vessel (blood vessel marked with * and necrosis marker with **). ×200 original magnification. **(f) **Tumours from bitransgenic MMTV-myr-Akt1, MMTV-c-ErbB2 mice have less apoptosis than tumours from MMTV-c-ErbB2 mice. Apoptosis was quantitated by activated caspase-3 immunohistochemistry. The number of cells staining positively for activated caspase-3 was divided by the total number of cells counted to generate the apoptotic rate. **(g) **Tumours from bitransgenic mice have a higher proliferation rate than tumours from MMTV-c-ErbB2 mice. Proliferation rate was determined by counting the number of mitotic figures in 10 500× magnification fields of view and the data is presented as the mean +/- standard deviation for three tumours of each genotype.

We have previously described the transgenic mice that express the activated myr-Akt1 transgene in the mammary gland which rarely develop tumours [11]. MMTV-c-ErbB2 (line 202) mice express the wild type rat *ERBB2 *gene in the mammary gland and have been an extensively used model of mammary carcinomas [20-22]. To determine whether myr-Akt1 could accelerate mammary tumourigenesis, we crossed MMTV-c-ErbB2 (line 202) mice [6] with MMTV-myr-Akt1 mice [11]. The MMTV-c-ErbB2 female mice developed single focal mammary tumours with a mean latency of 231 days (Figure 1a).

The bitransgenic MMTV-c-ErbB2, MMTV-myr-Akt1 mice developed single focal mammary tumours with a mean latency of 114 days, meaning the bitransgenic animals develop mammary tumours twice as fast as the MMTV-c-ErbB2 mice (Figure 1a). A total of 30 MMTV-c-ErbB2 mice and 22 bitransgenic mice were used to calculate mean tumour latency.

Tumours arising in the MMTV-c-ErbB2 mice usually display mutations in the wild type rat *c-ERBB2 *transgene resulting in a constitutively activated form of ErbB2 [18]. One possible means by which expression of myr-Akt1 could accelerate tumourigenesis in the MMTV-c-ErbB2 mice would be to bypass the apparent requirement for mutation of rat *c-ERBB2*. However, analysis of the tumours that appeared in the bitransgenic mice indicated that these tumours also contain the activating mutations in the rat *c-ERBB2 *allele (data not shown).

Tumour histology was evaluated using haematoxylin and eosin stained sections. The histology of the c-ErbB2 tumours was consistent with previous descriptions: they are solid tumours composed of uniformly sized and shaped cells with small stroma and no evidence of myoepithelial cells [6,23] (Figure 1b). Staining of tumours from the bitransgenic animals revealed two different types of tumours: those similar to c-ErbB2 tumours with a solid, uniform architecture (Figure 1c); and the second type of tumour demonstrated necrosis in areas of the tumour 10 to 20 cells away from vasculature, consistent with a tumour that outgrows its blood supply (Figure 1d, e). Very little necrosis is ever observed in tumours derived from MMTV-c-ErbB2 mice.

After initial detection, tumour volumes were determined by measuring the tumour dimensions with calipers to estimate tumour volume (volume = (length × width × width)/2). A comparison of tumour growth in mice of both genotypes revealed that tumour volume increased two to three times faster in the bitransgenic mice compared with tumours in the MMTV-c-ErbB2 transgenic mice (data not shown). The increased growth rate of tumours in the bitransgenic mice could result from either increased proliferation, decreased apoptosis or both. Measurement of the apoptotic rates in both tumour types by activated caspase-3 immunohistochemistry demonstrates that the tumours from the bitransgenic animals have an apoptotic rate half that of the tumours derived from the MMTV-c-ErbB2 animals (Figure 1f). The rate of proliferation, determined by quantitating the number of mitotic figures in tumour sections, demonstrated that tumours from bitransgenic animals had a higher proliferation rate than tumours from MMTV-c-ErbB2 animals (Figure 1g). Thus, the bitransgenic animals rapidly develop mammary tumours with a low rate of apoptosis and high rate of proliferation compared with the tumours from MMTV-c-ErbB2 animals, and at least half of these bitransgenic tumours exhibit extensive necrosis.

**Figure 2:**
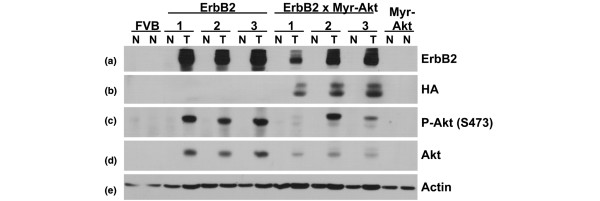
Tumours from bitransgenic MMTV-myr-Akt1, MMTV-c-ErbB2 mice and MMTV-c-ErbB2 mice overexpress ErbB2 and phosphorylated Akt. Tumour (T) and normal mammary tissue (N) were harvested from tumour-bearing mice. Tumours from three different bitransgenic MMTV-myr-Akt1, MMTV-c-ErbB2 mice and three different MMTV-c-ErbB2 mice are represented with normal mammary tissue from the same animal. Additionally, normal mammary tissue was collected from control FVB and MMTV-myr-Akt1 mice and analysed as a control. Equal amounts of total protein was loaded per lane to a 10% sodium dodecyl sulfate polyacrylamide gel, transferred to polyvinylidene difluoride and probed with the following antibodies: **(a) **anti-ErbB2; **(b) **anti-HA to detect the HA epitope-tagged myr-Akt1 transgene; **(c) **anti-phospho-Akt (Ser473); **(d) **anti-pan-Akt; and **(e) **anti-β-actin to demonstrate equal loading of the gel.

The expression of the transgenes was examined at the protein level in tumours (T) and normal mammary gland control (N) taken from tumour-bearing mice of both the MMTV-c-ErbB2 and MMTV-c-ErbB2, MMTV-myr-Akt1 genotypes. Mammary glands from FVB mice and MMTV-myr-Akt1 mice were used as controls. Immunoblotting with an anti-ErbB2 antibody demonstrated that the level of ErbB2 protein was dramatically increased in tumours of both origins compared with normal mammary tissue from the same mouse, from FVB mice or from myr-Akt1 transgenic mice (Figure 2a).

Expression of the HA-tagged myr-Akt1 transgene was only detected in tumour tissue from the bitransgenic animals (Figure 2b). The myr-Akt1 transgene in the bitransgenic tumours was phosphorylated at Ser473, indicating enzymatic activity, and can be distinguished from endogenous Akt because the myr-Akt1 transgene has a higher molecular weight (Figure 2c). Akt was also phosphorylated in c-ErbB2 tumours, consistent with previously published data [8] (Figure 2c). Immunoblot using anti-pan-Akt antibody demonstrates expression of endogenous Akt in both tumour types with the c-ErbB2 tumours expressing more Akt than the bitransgenic tumours (Figure 2d). An immunoblot with anti-β-actin antibody demonstrates equal sample loading (Figure 2e). All immunoblot data presented in the present study is representative of all tumour and normal gland pairs examined (n = 5 for c-ErbB2 and n = 7 for bitransgenic). The different levels of proteins expressed in tumour tissue versus normal tissue is probably due to both a difference in selective pressures brought on by tumourigenesis and a dramatic increase in epithelial cell content in tumour tissue: the virgin mammary gland is predominantly adipocytes and tumour tissue is predominantly epithelial in nature.

### Diminished overexpression and activation of EGFR family members in tumours from bitransgenic animals

**Figure 3:**
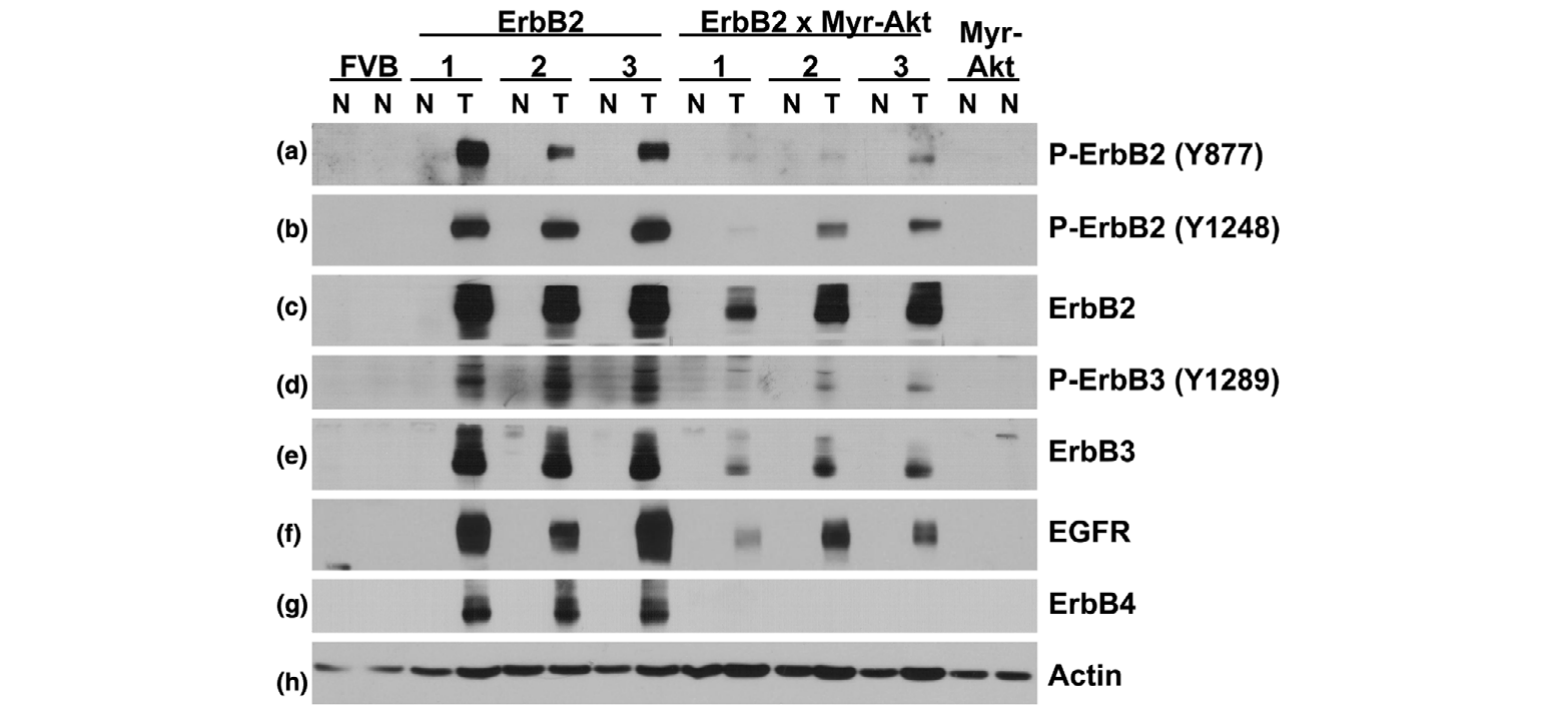
Expression and activation of EGF receptor tyrosine kinase family members is decreased in tumours from bitransgenic MMTV-myr-Akt1, MMTV-c-ErbB2 mice compared with tumours from MMTV-c-ErbB2 mice. Lysates from tumour (T) and normal mammary tissue (N) were used for immunoblot analysis using the following antibodies: **(a) **anti-phospho-ErbB2 (Tyr877); **(b) **anti-phospho-ErbB2 (Tyr1248); **(c) **anti-ErbB2; **(d) **anti-phospho-ErbB3 (Tyr1289); **(e) **anti-ErbB3; **(f) **anti-EGFR; **(g) **anti-ErbB4; and **(h) **anti-β-actin to demonstrate equal loading of the gel.

Mammary tumours from transgenic mice expressing activated mutants of ErbB2 also express elevated levels of total and tyrosine-phosphorylated ErbB2 and ErbB3 [7]. As previously shown in Figure 2, ErbB2 was increased in tumour lysates from both MMTV-c-ErbB2 and bitransgenic mice compared with normal tissue (Figure 3c). The extent of activating phosphorylation of ErbB2 was lower in the bitransgenic tumours than in the c-ErbB2 tumours, demonstrated by using phosphospecific antibodies to two different phosphorylation sites in ErbB2, Tyr877 and Tyr1248 (Figure 3a, b). Phosphorylation of Tyr877 is mediated by Src and contributes to the activation of the ErbB2 tyrosine kinase, and Tyr1248 is an autophosphorylation site [24,25]. Increased levels of ErbB3 (Figure 3e), Tyr1289-phosphorylated ErbB3 (Figure 3d), epidermal growth factor receptor (EGFR) (Figure 3f) and ErbB4 (Figure 3g) were readily detected in the c-ErbB2 tumours compared with normal tissue from the same mice.

While increased levels of EGFR and ErbB3 protein could be detected in the bitransgenic tumours when compared with normal tissue, the extent of the increase was dramatically reduced compared with that observed in the c-ErbB2 tumours. Most importantly, the phosphorylation of ErbB3 was very low at Tyr1289 (Figure 3d), which is an important phosphorylation site for PI3K recruitment and activation [26]. ErbB4, the sole member of the EGFR family with an expression that bears positive prognostic value to breast cancer patients and suppresses proliferation and promotes apoptosis [27,28], was not detected in the bitransgenic tumours (Figure 3g). These data suggest that expression of activated Akt1 in the MMTV-c-ErbB2 transgenic mice alters the requirement for overexpression and activation of ErbB3 and other EGFR family members in mammary tumours induced by ErbB2.

### Diminished activation of signalling downstream of ErbB2 in bitransgenic tumours

**Figure 4:**
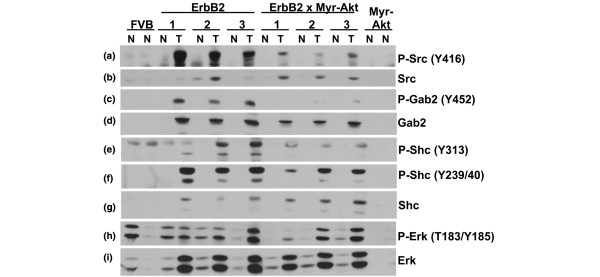
Tumours from MMTV-myr-Akt1, MMTV-c-ErbB2 mice have decrease tyrosine kinase signalling compared with tumours from MMTV-c-ErbB2 mice. Lysates from tumour (T) and normal mammary tissue (N) were used for immunoblot analysis using the following antibodies: **(a) **anti-phospho-Src (Tyr416); **(b) **anti-Src; **(c) **anti-phospho-Gab2 (Tyr452); **(d) **anti-Gab2; **(e) **anti-phospho-Shc (Tyr313); **(f) **anti-phospho-Shc (Tyr239/240): **(g) **anti-Shc; **(h) **anti-phospho-Erk (corresponding to Erk2 phosphorylation at Thr183 and Tyr185); and **(i) **anti-Erk.

Mammary tumours expressing c-ErbB2 have been found to possess elevated levels of Src tyrosine protein kinase activity [29,30]. The Src protein binds to phosphotyrosine residues in the cytoplasmic tail of ErbB2, resulting in Src phosphorylation at Tyr416 and catalytic activation [31,32]. The amount of Tyr416-phosphorylated Src present in the c-ErbB2 tumours was dramatically increased in comparison to the bitransgenic tumours and normal tissue (Figure 4a), even though the bitransgenic tumours express more total Src than the c-ErbB2 tumours (Figure 4b). The reduced Src activation indicated by low Tyr416 phosphorylation levels in the bitransgenic tumours is corroborated by the reduced phosphorylation of ErbB2 at Tyr877 (Figure 3a), which is mediated by Src [24,25]. The reciprocal positive regulation of Src by ErbB2 and ErbB2 by Src is strongly diminished in the bitransgenic tumours (in addition to the reduced activation of ErbB3) indicating a general decline in plasma membrane tyrosine kinase signalling in the bitransgenic tumours.

The lack of activation/overexpression of EGFR family members and the lack of activation of Src in the bitransgenic tumours suggested that signalling mediated by secondary signalling molecules might also be attenuated in the bitransgenic tumours when compared with c-ErbB2 tumours. Gab2 is a scaffolding protein that can recruit the p85 subunit of PI3K when it is phosphorylated at Tyr452 [33] which in turn activates PH-domain containing proteins such as Akt [34]. The levels of Gab2 protein are elevated in both c-ErbB2 tumours and bitransgenic tumours compared with normal gland controls (Figure 4d), although the level is slightly higher in c-ErbB2 tumours than bitransgenic tumours. The amount of Tyr452-phosphorylated Gab2 is dramatically higher in the c-ErbB2 tumours compared with the bitransgenic tumours (Figure 4c) suggesting that expression of activated Akt1 in the bitransgenic animals attenuates phosphorylation of Gab2 and subsequent docking of PI3K, which occurs in c-ErbB2 animals.

It has recently been demonstrated that another secondary signalling molecule, ShcA, is required for ErbB2-mediated tumourigenesis and phosphorylation of Tyr313 in ShcA may be important for tumour cell survival, while phosphorylation of Tyr239/240 may be important for tumour vascularisation [35]. c-ErbB2 tumours and bitransgenic tumours both demonstrate expression of ShcA (Figure 4g), particularly the p52 and the p46 isoforms while the p66 isoform was barely detectable. Both tumour types also appear to have similar levels of Tyr239/240-phosphorylated ShcA (with the bitransgenic tumours having slightly less phosphorylation than the c-ErbB2 tumours), indicating that activities regulated by phosphorylation of this site (perhaps in angiogenesis) are important in both tumour types (Figure 4f). However, the difference in phosphorylation of ShcA at Tyr313 is more dramatic between the two tumour types (Figure 4e), with the c-ErbB2 tumours having more ShcA phosphorylated at Tyr313 than the bitransgenic tumours suggesting that activities regulated by phosphorylation of this site (cell survival) may be less important in the tumours that express activated Akt1.

Oncogenic signalling often activates the Erk pathway, which is a known mediator of cell proliferation, cell survival, angiogenesis and cell migration (reviewed in [36]). Dual phosphorylation of Erk1 and Erk2 (corresponding to Thr183 and Tyr185 of human Erk2) in the activation loop results in catalytic activation of kinase activity [37]. Despite the attenuation of several signalling events in the bitransgenic tumours when compared with the c-ErbB2 tumours as discussed above, both tumour types maintain similar levels of phosphorylated/activated Erk (Figure 4h) indicating that transgenic activation of Akt1 does not bypass Erk signalling. Normal gland controls demonstrate various levels of Erk activation.

### Expression/activation of cell cycle control proteins in bitransgenic tumours

**Figure 5:**
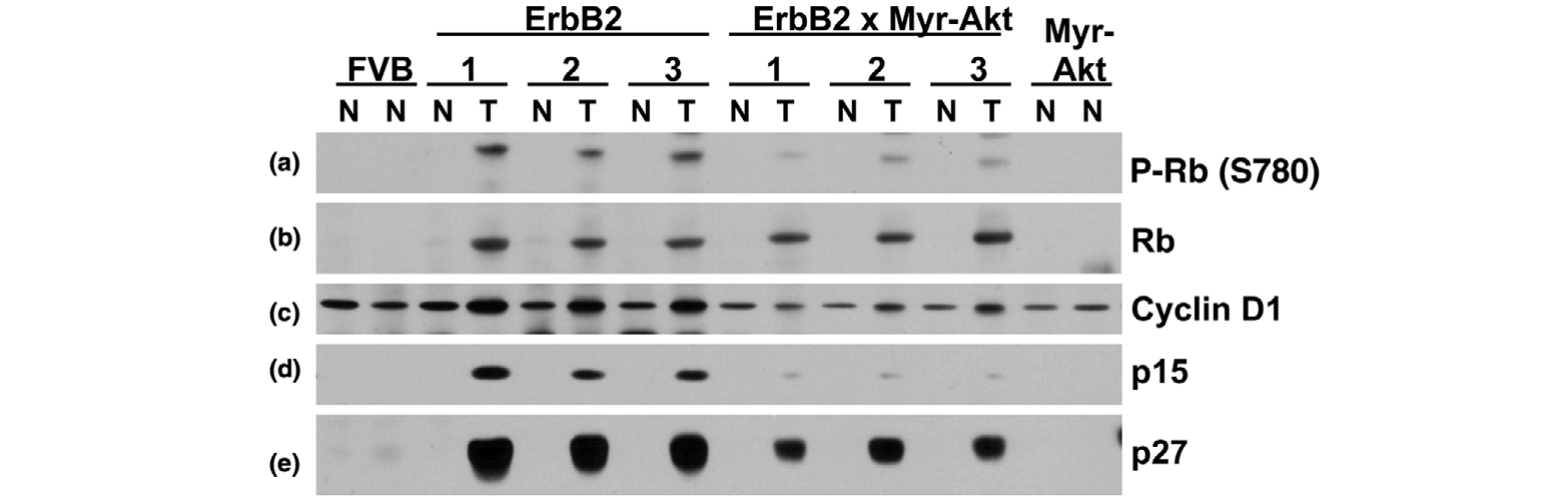
MMTV-c-ErbB2 tumours demonstrate different cell cycle proteins than tumours from bitransgenic MMTV-myr-Akt1, MMTV-c-ErbB2 mice. Lysates from tumour (T) and normal mammary tissue (N) were used for immunoblot analysis using the following antibodies: **(a) **anti-phospho-Rb (Ser780); **(b) **anti-Rb; **(c) **anti-cyclin D1; **(d) **anti-p15; and **(e) **anti-p27.

The decrease in the tumour latency observed in the bitransgenic tumours would predict that there would be a change in the expression/modification of cell cycle control proteins in the bitransgenic tumours compared with the c-ErbB2 tumours. Phosphorylation of Rb by active cyclin-dependent kinases inactivates Rb activity and is an important regulatory step in cell cycle entry [38,39]. Immunoblot analysis with a phospho-specific antibody against Rb (Ser780) demonstrated Rb phosphorylation in tumour lysates from both genotypes of mice and not in the normal tissue from the same mice (Figure 5a). The extent of Rb phosphorylation was greater in the c-ErbB2 tumours than in the bitransgenic tumours. This is surprising because the bitransgenic tumours have a shorter tumour latency and greater mitotic index which we predicted would correlate with more Rb phosphorylation and a more active cell cycle. However, Hutchinson *et al. *observed a similar phenomenon in their study: the NDL2 tumours (activated ErbB2) demonstrated a higher level of Ser780-phosphorylated Rb than the bitransgenic NDL2/Akt1DD tumours even though the bitransgenic animals had a shorter tumour latency [15]. The total amount of Rb is almost the same in all tumours examined indicating differences observed in Rb phosphorylation are not in fact due to differences in total Rb levels (Figure 5b).

We also examined a second cell cycle control protein, cyclin D1. The D-type cyclins are synthesised during the G1 phase of the cell cycle after stimulation of cells with growth factors that stimulate cell cycle entry and are required for cell cycle progression [40]. Immunoblot analysis revealed cyclin D1 in all samples with the bitransgenic tumours having a higher level of cyclin D1 than normal tissue, but the c-ErbB2 tumours containing more cyclin D1 than the bitransgenic tumours (Figure 5c). This data correlates with the Rb phosphorylation data which is consistent with the role of cyclin D1 in positively regulating cyclin dependent kinase activity and phosphorylation of Rb [40].

The bitransgenic tumours have a shorter latency than the c-ErbB2 tumours, but contain less phosphorylated Rb and less cyclin D1, which led us to examine some cell cycle inhibitors. p15 (INK4b), p27 (Kip1) and p21 (Cip1) are all inhibitors of cell cycle progression [41]. Immunoblot analysis for p15 demonstrated high expression in the c-ErbB2 tumours, a lack of expression in all normal glands and very faint expression in the bitransgenic tumours (Figure 5d). The expression profile of p27 was similar with a lack of expression in all normal tissue, high expression in c-ErbB2 tumours and slightly lower level of expression in the bitransgenic tumours (Figure 5e). We were unable to detect p53 and p21 in any of the tumour and normal mammary tissue samples, though both were present in an irradiated control gland (data not shown). While c-ErbB2 tumours contain more positive markers of the cell cycle than the bitransgenic tumours (phosphorylated Rb and cyclin D1), they also express more negative regulators of the cell cycle (p15 and p27), which likely serves to balance cell cycle progression.

### An elevated metabolic phenotype in bitransgenic tumours compared with MMTV-c-ErbB2 tumours

**Figure 6:**
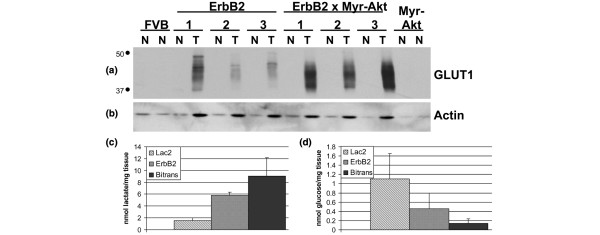
Metabolic activity is elevated in tumours from bitransgenic MMTV-myr-Akt1, MMTV-c-ErbB2 mice compared with tumours from MMTV-c-ErbB2 mice. Increased expression of the GLUT1 glucose transporter in bitransgenic MMTV-myr-Akt1, MMTV-c-ErbB2 tumours compared with the MMTV-c-ErbB2 tumours. Lysates from tumour (T) and normal mammary tissue (N) were used for immunoblot analysis using a polyclonal antibody against the c-terminus of GLUT1 **(a) **and actin-loading control **(b)**. Intratumour concentration (nmol/mg tissue) of lactate **(c) **and glucose **(d) **in mammary glands at day 2 of lactation (L2), tumours from MMTV-c-ErbB2 and bitransgenic MMTV-myr-Akt1, MMTV-c-ErbB2 mice (all n = 5) calculated from ^1^H-magnetic resonance spectroscopy.

One of the hallmarks of tumours and tumour cell lines is the elevated level of glucose transport to support the high glycolytic rate of tumour cells [42,43]. Myr-Akt1 can stimulate the translocation of the GLUT1 glucose transporter to the cell surface of lymphoid cells cultured in the absence of growth factors as well as increase hexokinase activity, glucose consumption and lactate production, which suggests that Akt stimulates aerobic glycolysis and produces the so-called 'Warburg effect' [44,45]. Immunoblot analysis demonstrates elevated levels of GLUT1 (the major glucose transporter in cancer cells) in lysates from mammary tumours from both the MMTV-c-ErbB2 and the bitransgenic mice compared with levels in normal glands. However, GLUT1 protein expression was greater in the bitransgenic tumours (Figure 6a). Cell-line transfection using GLUT1 cDNA validates the elongated band identified by GLUT1 immunoblot and the reason for this elongated band is probably due to glycosylation of GLUT1 (data not shown).

To determine whether the increased level of GLUT1 correlated with an increase in glycolysis (increased glucose consumption and increased lactate production), we used MRS to quantitate lactate concentrations in extracts prepared from tumours because lactate is the end product of glycolysis. The mammary tumours examined in this study are of epithelial cell origin, so the use of mammary tissue from day two of lactation (L2) as a control tissue allows for the comparison of normal epithelium with tumour epithelium, whereas the normal virgin mammary gland is composed mostly of adipocytes [46]. Compared with normal L2 tissue, there was a four-fold increase in the amount of lactate in the MMTV-c-ErbB2 tumours and a six-fold increase in the bitransgenic tumours (Figure 6c) (p < 0.00001 between L2 and both tumours and p < 0.02 between ErbB2 and bitransgenic tumours, all n = 5). This increase in tumour glycolytic activity was accompanied by significantly decreased intratumuor concentrations of glucose: 1.10 nmol/mg in the L2 mammary gland, 0.46 nmol/mg in ErbB2 tumours (p < 0.01) and 0.14 nmol/mg in bitransgenic tumours (p < 0.0004) (Figure 6d). This indicates high glucose utilisation due to the increased activity of glycolytic enzymes. Thus, the bitransgenic tumours appear to consume more glucose and secrete more lactate than the c-ErbB2 tumours or the normal epithelial cell-enriched lactating mammary gland.

## Discussion

We have demonstrated that expression of activated myr-Akt1 in MMTV-c-ErbB2 mice accelerates mammary tumourigenesis. Hutchinson *et al. *used a similar mammary tumourigenesis model using MMTV-NDL2-5 animals (which express activated ErbB2 in the mammary gland) and compared them with bitransgenic MMTV-NDL2-5, MMTV-Akt1DD animals (which express activated ErbB2 and an activated Akt1 gene that contains phosphomimetic mutations of Ser473 and Thr308) [15]. They found that expression of activated Akt1 accelerated ErbB2-induced tumourigenesis in their NDL2 model. The NDL2/Akt1DD bitransgenic tumours were more differentiated glandular tumours which expressed milk proteins. Expression of activated Akt1 in the MMTV-c-ErbB2 model did not induce more differentiated glandular tumours, but rather accelerated tumour formation often appearing to cause necrosis. Compared with the c-ErbB2 tumours, the bitransgenic tumours demonstrated half the amount of apoptosis and twice the amount of mitosis, suggesting that a decrease of apoptosis and increase in proliferation in the bitransgenic tumours contributed to faster tumour formation. Similar to Hutchinson *et al*., evaluation of apoptosis by TUNEL (terminal deoxynucleotidyl transferase dUTP nick end labeling) staining revealed almost no apoptotic cells in any of our tissue samples (data not shown), but evaluation of apoptosis by activated caspase-3 immunohistochemistry identified some apoptotic cells.

ErbB3, the major EGFR family member that activates the PI3K/Akt pathway [47], is required for ErbB2-induced cancer cell proliferation, transformation and colony formation *in vitro*[8,48]. There is a functional interaction between ErbB2 and ErbB3 in the MMTV-c-ErbB2 mouse model [8]. Activated Src is important for efficient ErbB2/ErbB3 heterocomplex formation and full activation of the ErbB2 kinase domain [24]. We have demonstrated that tumourigenesis in the MMTV-c-ErbB2 model proceeds with reduced activation of ErbB2, ErbB3, Src, Gab2 and Shc when activated Akt1 is expressed by transgene. Akt is often activated in cancer cells by the activating mutation of PI3K [49], inactivation of PTEN [50] and, a recently demonstrated mechanism, by mutation of Akt itself [51]. Our results demonstrate that Akt activation by any of these means may lead to less dependency on ErbB2/ErbB3 and Src signalling without inhibiting tumourigenesis. It is not clear whether the diminished activation of these signalling molecules reflects a reduced need for them to be activated due to the presence of activated Akt or a negative feedback loop by which activated Akt can suppress activation of ErbB3-dependent signalling.

Two recent reports studying tumourigenesis in MMTV-ErbB2 mice on an Akt1-/- background highlight the importance of Akt1 in ErbB2-induced tumourigenesis [52,53]. Both groups used activated ErbB2 models of tumourigenesis in Akt1-/- and Akt+/+ backgrounds and demonstrated the importance of Akt1 in mediating ErbB2-induced tumourigenesis: mice lacking Akt1 either failed to develop tumours or tumourigenesis was delayed. However, while Akt1 is required for efficient tumour formation in MMTV-ErbB2 mouse models, activation of Akt1 alone is not sufficient for mammary tumourigenesis [11,12] indicating that other pathways downstream of ErbB2 activation are important for tumour formation.

The Erk pathway is activated in many types of cancer and can be activated by numerous oncogenic signals, including ErbB2 (Figure 4h) [36]. The bitransgenic tumours used in the current study maintained Erk activation (despite the loss of numerous other signalling events), which suggests that Erk signalling is necessary in these tumours. This may be one explanation for why Akt is necessary for ErbB2 tumourigenesis [52,53], but is not sufficient for mammary tumourigenesis [11,12]: activation of Akt alone may fail to activate Erk.

Most tumour cells rely on increased glycolysis, even in the presence of available oxygen (the Warburg effect). It has been shown that p53, HIF-1 (hypoxia inducible factor 1), c-Myc as well as Akt can all upregulate glycolytic enzymes (often through inhibition of the mitochondrial tricarboxylic acid cycle) to trigger increased tumour cell glucose consumption [54]. The increased levels of lactate and reduced concentrations of glucose in both types of tumours in the present study is consistent with an increased glycolytic rate in the tumours, but the bitransgenic tumours contained more lactate and more GLUT1 than the c-ErbB2 tumours. This led to the hypothesis that activation of Akt1 induced aerobic glycolysis in these mammary carcinomas. This may offer another explanation for the accelerated tumour onset in the bitransgenic animals because glycolysis, while inefficient in terms of wasting the full oxidative potential of glucose, is efficient at producing ATP and also generating metabolic byproducts necessary for membrane synthesis [55].

Human breast carcinomas overexpress ErbB2 in 20% to 30% of cases and patients with this type of tumour bear a poor prognosis and are currently treated with trastuzumab, a humanised monoclonal antibody. Trastuzumab treatment significantly boosts the prognosis for patients with breast tumours that overexpress ErbB2, but the response rate for these patients is about 50% to 60% [56]. Nagata *et al. *demonstrated that loss of the PTEN tumour suppressor (which induces Akt activation) predicts resistance to trastuzumab treatment [57]. We predict that our bitransgenic animals represent a model of a patient who would be resistant to trastuzumab. Tumours from bitransgenic animals overexpress ErbB2, making them candidates for ErbB2-targeted therapy, but drugs which molecularly target ErbB2 (such as trastuzumab) or ErbB2/ErbB3 heterodimers (such as pertuzumab) may be futile because signalling downstream of ErbB2/ErbB3 is already attenuated in bitransgenic animals. If activation of Akt1 in human tumours (which can occur by loss of PTEN function or activating mutation of either PI3K or Akt) is critical to ErbB2-positive breast cancer cells becoming trastuzumab resistant, then evaluation of the activation status of Akt may assist in deciding the prognosis and treatment strategy for breast cancer patients as compared with the current screening that only identifies amplification of ErbB2. Additionally, therapies which molecularly target the Akt pathway may be critical to overcome trastuzumab resistance.

## Conclusion

Expression of activated Akt1 in the mammary gland of MMTV-c-ErbB2 mice accelerates tumourigenesis and attenuates signalling events sometimes thought to be critical to tumourigenesis. The bitransgenic tumours also have an accelerated glucose metabolism. Our studies suggest that tumours that overexpress ErbB2 which activate Akt by means of mutating PI3K, PTEN or Akt (rather than via ErbB2/ErbB3 activation of PI3K) may be resistant to ErbB2-targeted therapies. Therefore, therapies which molecularly target signalling events downstream of ErbB2, such as those mediated by Akt, may prove to be valuable.

## Abbreviations

ANOVA = analysis of variance; Akt1DD = constitutively active Akt1 mutant bearing two phospho-mimetic mutations; EGFR = epidermal growth factor receptor; ERK = extracellular-signal regulated kinase; FVB = inbred mouse strain which was exclusively used in this study; Gab2 = Grb2 associated binding protein 2; GLUT1 = glucose transporter 1; L2 = lactation day 2; HIF1 = Hypoxia inducible factor 1; MMTV = mouse mammary tumour virus; MRS = magnetic resonance spectroscopy; Myr-Akt1 = constitutively active Akt1 mutant bearing the myristolation sequence from Src; NMR = nuclear magnetic resonance; PI3K = phosphatidyl inositol-3 kinase; PTEN = phosphatase and tensin homologue deleted on chromosome 10; PVDF = polyvinylidene difluoride; Rb = retinoblastoma; SD = standard deviation; SDS-PAGE = sodium dodecyl sulfate polyacrylamide gel electrophoresis; Shc = Src homology 2 domain-containing transforming protein C1; TMSP = trimethylsilyl propionic-2,2,3,3,-d4 acid.

## Competing interests

The authors declare that they have no competing interests.

## Authors' contributions

CDY performed the immunoblot analyses, interpreted data, assembled the figures and drafted the manuscript. EN quantitated apoptosis, performed *ERBB2 *mutation analysis and assisted with preliminary studies. AL maintained the animal colony including breeding, genotyping, tumour palpation and tissue harvest. NJS performed MRS analysis and interpreted metabolic data. SMA conceived the study and directed the research. NJS and SMA critically reviewed the manuscript, which was approved by all authors.
